# Supplementary material for: Italian Version of the Pittsburgh Rehabilitation Participation Scale: Psychometric Analysis of Validity and Reliability
Source: Brain Sci. 2021 May 13;11(5):626. doi: 10.3390/brainsci11050626 (PMC8153139; doi:10.3390/brainsci11050626)
Supplement: Supplementary file 1 [file brainsci-11-00626-s001.zip › brainsci-1174557-supplementary.pdf]

Supplementary Material

Table S1. On the left the original PRPS validated in English, on the right the proposed Italian version of PRPS.

**Original English version of PRPS**		**Proposed Italian version of PRPS**	
Istructions to therapist	For each therapy session, please circle one of each of the following to assess the patient's participation (effort and motivation as perceived by you) in the therapy session. Please rate as follows: (see Note below)	Istruzioni per il terapista	Per ogni sessione di terapia, si prega di individuare uno dei seguenti elementi per valutare la partecipazione del paziente (sforzo e motivazione percepita da voi) alla sessione di terapia. Si prega di valutare come segue: (vedi le note sotto)
None	Patient refused entire session, or did not participate in any exercises in session.	Per niente	Il paziente ha rifiutato l'intera seduta, o non ha partecipato ad alcun esercizio in seduta.
Poor	Patient refused or did not participate in at least half of session.	Mediocre	Il paziente ha rifiutato o non ha partecipato ad almeno metà della seduta.
Fair	Patient participated in most or all of exercises*, but did not show maximal effort or finish most exercises*, or required much encouragement to finish exercises*.	Discreto	Il paziente ha partecipato alla maggior parte o a tutti gli esercizi*, ma non ha mostrato il massimo sforzo o non ha concluso la maggior parte degli esercizi*, o ha richiesto molto incoraggiamento per terminarli*.
Good	Patient participated in all exercises* with good effort and finished most but not all exercises* and passively followed directions (rather than actively taking interest in exercises* and future therapy).	Buono	Il paziente ha partecipato a tutti gli esercizi* con un buon sforzo e ha terminato la maggior parte ma non tutti gli esercizi* e ha seguito passivamente le indicazioni (piuttosto che interessarsi attivamente agli esercizi* e alla terapia futura).
Very good	Patient participated in all exercises* with maximal effort and finished all exercises, but passively followed directions (rather than actively taking interest in exercises* and future therapy).	Molto buono	Il paziente ha partecipato a tutti gli esercizi* con il massimo sforzo e ha terminato tutti gli esercizi, ma ha seguito passivamente le indicazioni (piuttosto che interessarsi attivamente agli esercizi* e alla terapia futura).
Excellent	Patient participated in all exercises* with maximal effort, finished all exercises*, and actively took interest in exercises* and/or future therapy sessions	Eccellente	Il paziente ha partecipato a tutti gli esercizi* con il massimo sforzo, ha terminato tutti gli esercizi*, e attivamente si è interessato agli esercizi* e/o alle future sessioni terapeutiche.
Notes	* if patient was unable to attend therapy because of medical test, bed rest order, illness, or scheduling conflict, do not mark any score.<br>* in cases of doubt, choose the lower rating, e.g., "good" rather than "very good."	Note	* se il paziente non ha potuto partecipare alla terapia a causa di esami medici, ordine di riposo a letto, malattia, o di pianificazione dei conflitti, non segnare alcun punteggio.<br>* in caso di dubbio, scegliere il rating più basso, ad esempio, "buono" piuttosto che "molto buono".
